# Seasonal patterns of suicides over the period of socio-economic transition in Lithuania

**DOI:** 10.1186/1471-2458-6-40

**Published:** 2006-02-22

**Authors:** Ramune Kalediene, Skirmante Starkuviene, Jadvyga Petrauskiene

**Affiliations:** 1 Department of Social Medicine, Kaunas University of Medicine, Kaunas, A. Mickeviciaus St 9, LT-44307 Kaunas, Lithuania

## Abstract

**Background:**

In Lithuania, suicides are a grave public health problem, requiring more extensive investigation. The aim of the study was to assess the seasonal variations of suicides in Lithuania throughout the years 1993–2002, describing patterns by gender, age and method of suicide.

**Methods:**

The study material consisted of all registered suicides (n = 16,147) committed throughout 1993–2002 in Lithuania. Smoothed trends were inspected. The seasonal effect was explored using monthly ratio statistics and spectral analysis.

**Results:**

Suicides in Lithuania have a distinct annual rhythm with peaks in summer and troughs in December. The December frequencies fell by more than 23% in men and 30% in women, while June peak reached nearly 23% in men and July peak exceeded 29% in women, compare with the average levels, (p < 0.05). Hanging was the most common method of suicide both in men and women comprising up to 90% among all suicides in 1998–2002. Among different methods, only hanging suicides showed significant seasonal variations, especially in men. The seasonal amplitude has decreased over time.

**Conclusion:**

Substantial seasonal variations in suicides were associated with a high proportion of hanging. Extremely high suicide rates in Lithuania require further extensive studies and urgent preventive programs, taking into account the suggestions of this survey.

## Background

In Lithuania, suicide is one of the leading causes of death of able-bodied population, particularly men. A period of the major social and economic instability (1990–1994) was marked by dramatically increasing suicide mortality, reaching its highest ever-reported levels in 1994 in men (87.7 per 100,000) and 1995 in women (15.0 per 100,000). Although later suicides started to decline, they still remain among the highest in Europe [1]. Responding to suicides requires extensive research for understanding their possible determinants. The research that has been conducted in Lithuania has provided a better understanding of the wide range of associations of suicides with the place of residence, level of education, as well as some biologic processes, including environmental physical activity factors [2-6]. Inequalities in daily variations of deaths from suicide have also been analysed, demonstrating that the highest proportion of suicides in Lithuania occurs on Mondays and during the first days after the major public holidays [7]. Seasonality of suicides is an ubiquitous phenomenon, reported by researchers from different countries. Most of studies have found that suicide rates tend to peak during spring and summer [8-12]. Generally, a remarkably consistent pattern of seasonality with peak incidence in June in the Northern hemisphere and December in the Southern hemisphere is observed [13]. However, some of the researchers predict that the seasonality of suicides would disappear in the new millennium [14], while contradictory data do exist [11,15]. An understanding of the nature and causes of seasonality in suicides may have implications for health policy, by identifying those at particular risk and suggesting potentially avoidable risk factors.

The aim of our study was to assess the seasonal variations of suicides in Lithuania throughout the years 1993–2002, describing patterns by gender, age and method of suicide.

## Methods

Information on suicides for the period of 1993–2002 was derived from computerized database of Lithuanian Department of Statistics. These files contained records abstracted from death certificates. Determined cases of suicides (ICD-9: E950-959) and intentional self-harm (ICD-10: X60-X84) were included in the analysis. The study material consisted of all registered suicides (n = 16,147) committed from 1 January 1993 to 31 December 2002 in Lithuania. All cases were classified by gender, age, suicide method, year and month of death. Analysis of suicide rates rather than person-years was conducted. The size of the total Lithuanian population was nearly stable throughout the period of investigation, accounting for 3.2–3.7 million people. The whole study period was divided into two time periods: 1993 to 1997 and 1998 to 2002, in order to test the change in the amplitude over time. This time period was marked by dramatic social and political changes occurring in the country, usually called period of transition from highly centralized Soviet system to market economy. During the period of 1993–1997, there were 8,324 cases of suicides registered (6,864 among men and 1,460 among women), while in 1998–2002 7,823 persons committed suicides (6,455 men and 1,368 women). The autopsy rate in Lithuania is very high, thus the register of deaths can be considered to be reliable.

Due to the relative small numbers of suicides per month, there was increased variability in rates; thus monthly variations were examined after rates were smoothed with 3-month moving averages over two periods. Later, the monthly effect was evaluated by the ratio statistics, the null hypothesis being that the suicides in each group of methods are evenly distributed over the year. Amplitude of the monthly variations in suicides was presented as the ratio of observed to expected frequencies of suicides within each month of the decade under investigation and separately in two different periods with 95% confidence intervals (CI). The gender differences and seasonal distribution of suicides within each method and time period was tested with the Chi-squared test (χ^2^). Additionally, spectral analysis was used to get more detailed information about the cyclical patterns separately for the different periods, methods and genders [16]. The following definitions for seasons were used, taking into account different climatic conditions: winter (December-February), spring (March-May), summer (June-August), and autumn (September-November). The leap years and unequal numbers of days in a month were taken into account while performing statistical analysis. The statistical software package SPSS for Windows version 10.0 was used.

This study was based on official register, and thus no approval from the ethics committee was required.

## Results

**Figure 1:**
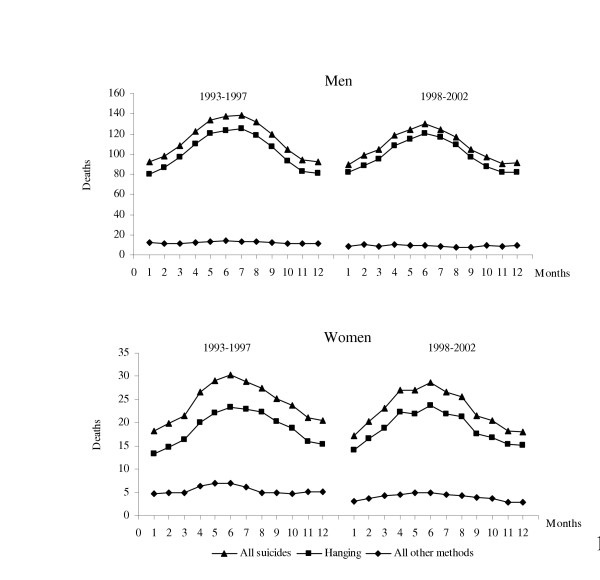
Smoothed trends in suicides.

Analysis of smoothed (three-point moving) averages in the variations of suicide suggested an obvious seasonal pattern both for men and women over two different periods under investigation (Figure 1). Deaths from suicide showed a decrease in the beginning and the end of the year and striking summer excess, especially in men. This was particularly notable for hanging suicides.

Monthly variations of suicides are presented in Table 1. There was an obvious peak in suicides observed both among men and women in May, June and July, with the greatest June frequencies for men in 1993–1997 and July for women in 1998–2002. Nevertheless, comparison of two time periods did not disclose statistically significant time changes in the monthly ratios of suicides. The Chi-squared test yielded χ^2 ^values of 14.02 (df = 11, p = 0.232) for men; and 8.68 (df = 11, p = 0.65) for women, respectively.

Table 2 shows the seasonal variations of suicides, expressed in percent deviations from the daily average per year. Seasonal pattern was obvious both in men and women, with the greatest decline from the daily average per year of suicides in winter. Whereby, the peak of suicides occurred in summer, and was statistically significant in men. Though for women the winter-trough was 23.5–24.2% in two periods under investigation, the results were not statistically significant mainly due to rather low frequencies. Nevertheless, over the entire period of the study, statistically significantly lower rates in winter were observed among women. Generally, the difference in seasonal distributions of suicides between men and women did not show statistical significance, with χ^2 ^= 5.009, (df = 3; p = 0.165).

Within all registered suicides in Lithuania throughout 1993–2002, hanging was the most frequent method. Of the total number of completed suicides, 89.4% of men and 77.3% of women who committed suicide during 1993–1997 chose hanging. Over the period of the study, the proportion of hanging increased statistically significantly and reached 91.7% in men and 82.6% in women in 1998–2002, (p < 0.05). The other predominant methods among men were firearms and explosives, while among women – poisoning by solid or liquid substances or gases. Self-inflicted poisoning was registered in every tenth women, while in men this method was uncommon (about 2%).

**Figure 2:**
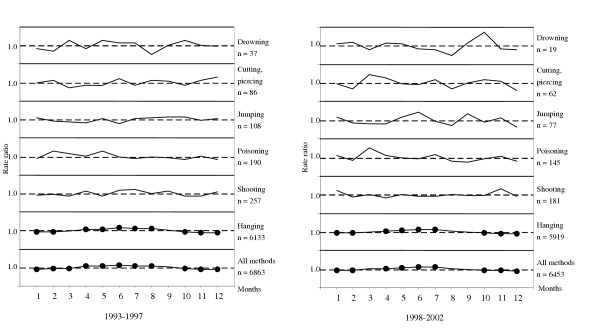
**The monthly ratios of suicides by method among Lithuanian men. **Statistically significant peaks are dark-dotted.

**Figure 3:**
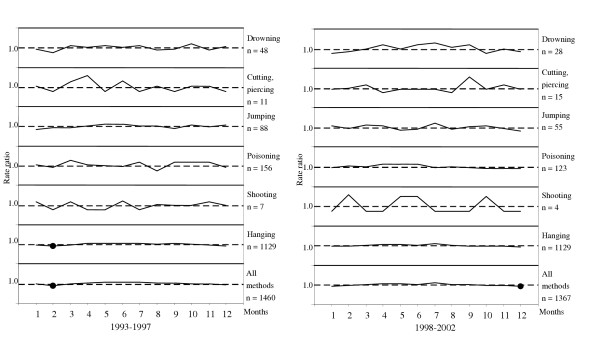
**The monthly ratios of suicides by method among Lithuanian women. **Statistically significant peaks are dark-dotted.

Further analysis has been undertaken to identify monthly distribution of suicides by gender and different methods. Figures 2 and 3 present the monthly ratios of suicides during two periods under investigation. Hanging suicides in men showed a distinct and statistically significant seasonality with the peak in summer months with the monthly ratios reaching 1.2 in June and July both in 1993–1997 and 1998–2002, whereas suicides due to other means did not disclose seasonality. For women, the only significant difference from the average (based on 95% CI) was observed in hanging in February 1993–1997 (the monthly ratio being 0.6). There was a suggestion of downturn in suicides at the end of the year by majority of other means, however, due to relatively low frequencies, the results from the monthly variation remained inconclusive.

More detailed analysis of monthly frequencies of hanging throughout the period of 1993–2002 showed a distinct peak in June and the trough in December, with the range varying between +24.4% to -24.5% among men, (p < 0.05, based on 95% CI). Among women, July peak exceeded the average rates by 34.0%, and December hanging frequencies were 30.6% below the average, (p < 0.05).

The analysis by age provided another insight in the monthly distribution of suicides. Statistically significant variation through months for men occurred in the age group of 20–64 years with excess mortality throughout April-August, and trough in October-February, while in the age group over 65, lower mortality rates occurred only in February (p < 0.05, based on 95% CI). For women, the only significant declines from the daily average per year were observed only in age group 20–64 in December and February. Nevertheless, there was no seasonality observed among those men and women who committed suicides up to the age of 20 and among women over 65 years.

Spectral analysis confirmed a single 12-month cycle both for men and women. The amplitude has decreased over time. In 1993–1997 it explained 72.3% of men and 40.9% of women total suicide variance, while in 1998–2002 this percentage declined to 53.4% in men and 35.9% in women, (p < 0.05). The declining amplitude of total suicide seasonality was paralleled by the decrease in the amplitude of hanging over the period under investigation: a single peak for the 12-month period with a decrease in explained variance from 74.0% to 58.7% in men and from 46.2% to 32.1% in women was observed, (p < 0.05). Seasonality of other methods of suicides was not statistically significant.

## Discussion

Before discussing findings of this study, it is important to consider reliability of the data and other potential limitations. According to the Lithuanian regulations, registration of all cases of unnatural deaths must be based on medico-legal investigation, and the autopsy rate covers nearly all such cases. However, despite the high degree of formalization in assessment of unnatural deaths, suicide numbers derived from mortality statistics might generally underestimate the real numbers to a certain extent. Suicide is very specific cause of death, which may be masked by other causes, such as accidents or undetermined causes of death, due to the sensitivity of society to such a diagnosis. Nevertheless, quantitative and qualitative analysis of the reliability of the statistics of suicide in the republics of the former Soviet Union during the period of 1970–1990 demonstrated that suicide mortality statistics were reliable in the Baltic republics [17]. The limitation of this study regards the possibility of incomplete counts, since a considerable number of suicide episodes does not result in death and were not included in the study. Although, at an aggregate level, it is impossible to assess the impact of particular factors on suicide, this type of surveys overcome sampling problems by using population-based data, over a long period of time, which is important in applying statistical analysis for monthly data separately for different suicide methods in both genders and drawing statistically significant results. Hence, the findings of this study may be helpful in developing in-depth research surveys and suicide prevention programs.

The pattern of seasonal variation in suicides in Lithuania displays similarities with that in other countries. Suicide seasonality differs greatly by suicide method. It is well known that violent suicides are more impulsive rather than premeditated, and they usually show considerable seasonality when compared to non-violent methods. This appears to be particularly true in the countries with a higher percentage of suicide by hanging, and strangulation [18]. Several studies have shown that violent suicides depict a seasonality with peaks in spring and summer [18,19]. Nevertheless, some surveys report that suicide seasonality is absent or weak in some violent methods [8]. Hanging, as the method of suicide has been historically popular in Lithuania. Already before 1940's hanging was the most common suicide method, though accounted for less than one third of all suicides [20]. This highly lethal method was gradually increasing in Lithuania during the last decades, reaching around 90% among all suicides in 1998–2002. Prevalence of particular methods of suicide differs by the country. Hanging is also the most frequent type of suicide in countries such as Germany, Israel, Slovenia, Hungary and Estonia [21-23], however the proportions of the reported hanging are considerably lower than in Lithuania.

The present study suggests the obvious seasonality for hanging among men with peaks in June of over 24%, and more than 24% lower December frequencies, while for women those fluctuations were even greater. Direct international comparisons of variation amplitudes are rather complicated due to different methodologies applied. Nevertheless, the seasonal variation in hanging in Lithuania is of bigger amplitude compare to the recent Finnish data [10]. Majority of studies report considerably greater suicide seasonality among men, explaining this fact by more frequent use of more violent methods among them [10,19,24]. Based on spectral analysis, the Lithuanian dataset supports this observation, suggesting greater hanging seasonality among men during two periods under investigation. Nevertheless, large seasonality, though declining over the decade, but still remaining strong was reported in women, with 32.1% explained variance in 1998–2002. This might be related to extraordinarily high proportion of hanging among Lithuanian women. However, the reasons of choosing this highly lethal method of suicide among Lithuanian women have never been investigated.

The findings from different countries report controversial changes in seasonality of suicides over time, generally suggesting the decrease in the amplitudes in the recent decades [14]. Lithuania also seems to belong to the group of countries with declining monthly variation of suicides throughout the last decade. Some authors conclude that stable or increasing suicide seasonality is characteristic to societies in transition [15]; nevertheless, contradictions do exist. Seasonality of suicide in Hungary decreased over the last decades, while some highly stable societies, like Australia report even increasing suicide seasonality [11,25]. The positive association of seasonality and the incidence of suicide are well known [25], therefore pronounced seasonality of suicides in a country with extremely high violent suicide rates should not be surprising.

There is an evidence about the meteorological, biological and physiological mechanisms underlying seasonality of suicides [2,26]. December decline and spring-summer increase in suicides is widely discussed in the literature [8,10,18]. The latitude and climatic factors, such as day length, daily temperature, daylight, and humidity may influence mood [27,28] and impulsive behaviour [29,30]. The monthly averages of humidity, ambient temperature, duration and intensity of sunlight are positively correlated with the number of monthly suicide attempts, while cloudiness and atmospheric pressure are negatively correlated [31]. Since the climatic conditions vary substantially between seasons in Lithuania, the effect of season on violent methods of suicides should be seen more clearly there than in countries nearer equator.

It has been suggested that seasonal vulnerability is biologically determined and associated with the circannual rhythms of central serotonin neurotransmission [26]. A combination of hopelessness with aggressive and impulsive behaviour, anhedonia is related to low brain serotonin levels and is a predictor of suicide [32]. In recent years, it has been demonstrated that serotonin production and serotonin-related medical and biological events (e.g., severity of migraine attacks, suicide rates, and blood coagulation) are related to environmental physical activity. Several studies have shown that suicide rates are inversely related to cosmophysical (i.e., solar and cosmic-ray) and geomagnetic activity [5,6]. Evidence have been provided that violent suicides and suicide attempts are higher among those with presence of depressive disorders [33]; while depressed suicides are known to display seasonal variations with peaks occurring in spring and summer [34]. Unfortunately, only limited data on the prevalence of depressions in Lithuania are available. The study on Lithuanian schoolchildren's self-reported explanations of suicidal ideation demonstrated that the most common reasons for suicidal inducements were depressing feelings and experiences [35]. Surveys on influence of depression on suicides in Lithuania, however, have not been accomplished yet, and is a subject of further research.

It is difficult to distinguish biometeorological and endogenous effects from the social and cultural factors. Sociological arguments assuming that a higher level of social tensions and thus the seasonal peak in the second quarter of the year was developed by Durkheim [36]. A more recent sociological approach to suicide seasonality labelled "broken promise theory" has been presented by Gabennesch [37]. Vulnerable individuals may be influenced by approaching holidays, because it tends to promote hopes or expectations in people that they will feel better after holidays than they did before. When these expectations are not met, the resulting disappointment may trigger a suicidal reaction [26]. However, the annual peak of suicides, reached before the end of usual summer vacations in Lithuania, points at other possible factors, occurring during summer months. Interestingly, the findings of this survey link to our previous report on daily variations of suicides in Lithuania, where Monday and after holidays peaks in suicides were presented. Although there were no individual data on alcohol consumption available for this survey, alcohol seemed to be strongly linked to weekly variations in mortality from suicides [7]. The reports of other authors support our suggestions [38]. Lithuania is a country that has been undergoing intensive social, political and economic transition over the last decade, which inevitably affected population of the country, requiring huge psychological adaptability efforts. The case for alcohol playing an important role in fluctuating mortality in Lithuania over the last decade of 20^th ^century has been widely discussed [7,39]. The binge drinking culture that exists in Lithuania and the growth of alcoholism is one of the most distressing social problems, directly related to the mortality from suicides. The recent Lithuanian forensic research data suggested that 53.3% of suicides were committed in the state of alcohol inebriation, with alcohol concentration exceeding 0.4‰. The proportion of suicides under alcohol inebriation reached the highest level in summer (60%) [40]. In Moscow, a study of seasonal variation in mortality also pointed at harmful consequences of alcohol consumption to address the observed summer peak [41]. Reliable data on seasonality in drinking pattern is not available for Lithuania, nevertheless, indirect observations suggest that alcohol is more commonly used during summer vacations, which might consequently contribute to a higher risk for destructive behaviours. However, the data available for this survey were not sufficient for implying alcohol as a possible causal factor for seasonality of suicides.

## Conclusion

The findings of this study demonstrate substantial seasonal variations in suicides with peaks in summer and troughs in December, which were associated with the extremely high proportions of hanging both among men and women. Based on spectral analysis, the amplitude of seasonal variations in suicides has declined over the period of 1993–2002. Although at an aggregate level it was impossible to determine the reasons of remarkable seasonal variations of suicides, enormously high suicide rates in Lithuania require further extensive studies and urgent preventive programs, taking into account the suggestions of this survey.

## Competing interests

The author(s) declare that they have no competing interests.

## Authors' contributions

RK substantially contributed to the design of the article, and to the interpretation of data. SS helped with the analysis of the data, and drafting the manuscript. JP participated in the design of the study, have been involved in drafting manuscript and revised it critically for important intellectual content. All authors read and approved the final version of the manuscript.

## Pre-publication history

The pre-publication history for this paper can be accessed here:



## Figures and Tables

**Table 1:** Monthly variations (%) and absolute numbers of suicides in Lithuania

Month	1993–1997		1998–2002		1993–2002	
	Men	Women	Men	Women	Men	Women
January	-17.3*	-12.1	-8.0*	-19.9	-12.8*	-15.9
	482	109	504	93	986	202
February	-14.3*	-33.9*	-14.3*	-17.0	-14.3*	-25.8*
	451	74	424	87	875	161
March	-8.0*	-8.1	1.3	7.7	-3.5	-0.5
	536	114	555	125	1091	239
April	12.9*	10.8	9.7*	19.3	11.4*	14.9
	637	133	582	134	1219	267
May	14.1*	21.8	17.9*	24.9	15.9*	23.3*
	665	151	646	145	1311	296
June	25.0*	26.7	20.3*	11.3	22.7*	19.2
	705	152	638	125	1343	277
July	18.4*	21.8	21.3*	36.9	19.8*	29.1*
	690	151	665	159	1355	310
August	16.5*	5.6	3.5	-0.9	10.2*	2.5
	679	131	567	115	1246	246
September	7.8	6.7	-1.6	-1.2	3.2	2.9
	608	128	522	111	1130	239
October	-13.2*	-3.2	-12.4*	-16.5	-12.8*	-9.6
	506	120	480	97	986	217
November	-19.5*	-10.8	-15.0*	-12.8	-17.3*	-11.8
	454	107	451	98	905	205
December	-22.8*	-27.4	-23.5*	-32.8*	-23.2*	-30.0*
	450	90	419	78	869	168

* – p < 0.05 compare to the daily average per year, statistics based on 95% confidence intervals.

**Table 2:** Seasonality (%) and absolute numbers of suicides in Lithuania

Season	1993–1997		1998–2002		1993–2002	
	Men	Women	Men	Women	Men	Women
Winter	-18.3*	-24.2	-15.3*	-23.5	-16.9*	-23.8*
	1383	273	1347	258	2730	531
Spring	6.3	8.2	9.6*	17.3	7.9*	12.6
	1838	398	1783	404	3621	802
Summer	19.9*	17.9	15.0*	15.8	17.5*	16.9
	2074	434	1870	399	3944	833
Autumn	-8.4*	-2.5	-9.7*	-10.2	-9.0*	-6.2
	1568	355	1453	306	3021	661

* – p < 0.05 compare to the daily average per year, statistics based on 95% confidence intervals.

## References

[B1] WHO/Europe, HFA Database, June 2004. http://www.who.dk/hfadb.

[B2] Stoupel E, Gabbay U, Petrauskiene J, Abramson E, Kalediene R, Sulkes J (2000). Heart-mood-death: the clinical expression of the cholesterol-serotonin controversy by the temporal distribution of death from coronary heart disease and suicide. J Clin Basic Cardiol.

[B3] Kalediene R (1999). Time trends in suicide mortality in Lithuania. Acta Psychiatr Scand.

[B4] Kalediene R, Starkuviene S, Petrauskiene J (2004). Mortality from external causes in Lithuania: looking for critical points in time and place. Scand J Public Health.

[B5] Stoupel E, Petrauskiene J, Kalediene R, Abramson E, Sulkes J (1995). Clinical cosmobiology: the Lithuanian study 1990–1992. Int J Biometerol.

[B6] Stoupel E, Kalediene R, Petrauskiene J, Starkuviene S, Abramson E, Israelevich P, Sulkes J (2005). Suicide-homicide temporal interrelationship, links with other fatalities, and environmental physical activity. Crisis.

[B7] Kalediene R, Petrauskiene J (2004). Inequalities in daily variations of deaths from suicide in Lithuania: identification of possible risk factors. Suicide Life Threat Behav.

[B8] Ajdacic-Gross V, Wang J, Bopp M, Eich D, Rossler W, Gutzwiller F (2003). Are seasonalities in suicide dependent on suicide methods? A reappraisal. Soc Sci Med.

[B9] Partonen T, Haukka J, Kaisa V, Hakko H, Pirkola S, Isometsa E, Lonnqvist J, Sarkioja T, Vaisanen E, Rasanen P (2004). Cyclic time patterns of death from suicide in northern Finland. J Affect Disord.

[B10] Räsänen P, Hakko H, Jokelainen J, Tiihonen J (2002). Seasonal variation in specific methods of suicide: a national register study of 20 234 Finnish people. J Affect Disord.

[B11] Rock D, Greenberg DM, Hallmayer J (2003). Increasing seasonality of suicide in Australia 1970–1999. Psychiatry Res.

[B12] Altmura C, VanGastel A, Piopi R, Mannu P, Maes M (1999). Seasonal and circadian rhythms in suicide in Cagliari, Italy. J Affect Disord.

[B13] Petridou E, Papadopoulos FC, Frangakis CE, Skalkidou A, Trichopoulos D (2002). A role of sunshine in the triggering of suicide. Epidemiology.

[B14] Yip PS, Chao A, Chiu CW (2000). Seasonal variation in suicides: diminished or vanished. Experience from England and Wales, 1982–1996. Br J Psychiatry.

[B15] Voracek M, Vintila M, Fisher ML, Yip PS (2002). Evidence for lack of change in seasonality of suicide from Timis County, Romania. Percept Mot Skills.

[B16] Gottman JM (1981). Time-series analysis.

[B17] Wasserman D, Varnik A (1998). Reliability of statistics on violent death and suicide in the former USSR, 1970–1990. Acta Psychiatr Scand Suppl.

[B18] Lester D (1999). Seasonal variation in suicide and the methods used. Percept Mot Skills.

[B19] Maes M, Cosyns P, Meltzer HY, De Meyer F, Peeters D (1993). Seasonality in violent suicide but not in nonviolent suicide or homicide. Am J Psychiatry.

[B20] Sopauskas J (1939). Morbidity in Lithuania on the basis of registration of deaths and communicable diseases Kaunas.

[B21] Schmidke A (1997). Perspective: suicide in Europe. Suicide Life Threat Behav.

[B22] Marušiè A (1999). Suicide in Slovenia: lessons for cross-cultural psychiatry. Int Rev Psychiatry.

[B23] Nachnam R, Yanai O, Goldin L, Swartz M, Barak Y, Hiss J (2002). Suicide in Israel: 1985–1997. J Psychiatry Neurosci.

[B24] Lester D, Frank ML (1988). Sex differences in the seasonal distribution of suicides. Br J Psychiatry.

[B25] Voracek M, Fisher ML, Yip PS, Zonda T (2004). Seasonality of suicide in Eastern Europe: A rejoinder to Lester and Moksony. Percept Mot Skills.

[B26] Preti A, Miotto P, De Coppi M (2000). Season and suicide: recent findings from Italy. Crisis.

[B27] Potkin SG, Zetin M, Stamenkovic V, Kripke DF, Bunney WE (1986). Seasonal affective disorder: prevalence varies with latitude and climate. Clin Neuropharmacol.

[B28] Lambert GW, Reid C, Kaye DM, Jennings GL, Esler MD (2002). Effect of sunlight and season on serotonin turnover in the brain. Lancet.

[B29] Maes M, Scharpe S, Verkerk R, D'Hondt P, Peeters D, Cosyns P, Thompson P, De Meyer F, Wauters A, Neels H (1995). Seasonal variation in plasma L-tryptophan availability in healthy volunteers. Relationships to violent suicide occurrence. Arch Gen Psychiatry.

[B30] Souetre E, Salvati E, Belugou JL, Douillet P, Braccini T, Darcourt G (1987). Seasonality of suicides: environmental, sociological and biological covariations. J Affect Disord.

[B31] Doganay Z, Sunter AT, Guz H, Ozkan A, Altintop L, Kati C, Colak E, Aygun D, Guven H (2003). Climatic and diurnal variation in suicide attempts in the ED. Am J Emerg Med.

[B32] Lader MH, Cowen PJ (2001). Depression. British Med Bulletin.

[B33] Astruc B, Torres S, Jollant F, Jean-Baptiste S, Castelnau D, Malafosse A, Courtet P (2004). A history of major depressive disorder influences intent to die in violent suicide attempters. J Clin Psychiatry.

[B34] Klim CD, Lesage AD, Seguin M, Chawky N, Vanier C, Lipp O, Turecki G (2004). Seasonal differences in psychopathology of male suicide completers. Compr Psychiatry.

[B35] Zemaitiene N, Zaborskis A (2004). Schoolchildren's self-reported explanations of suicidal ideation. Psichologija.

[B36] Durkheim E (1983). Der Selbstmord.

[B37] Gabennesch H (1988). When promises fail: a theory of the temporal fluctuations of suicide. Soc Forces.

[B38] Pirkola S, Isometsa E, Heikkinen M, Lobbgvist J (1997). Employment status influences the weekly patterns of suicide among alcohol misusers. Alcohol Clin Exp Res.

[B39] Leon D, Chenet L, Shkolnikov VM, Zakharov S, Shapiro J, Rakhmanova G, Vassin S, McKee M (1997). Huge variation in Russian mortality rates 1984–1994: artefact, alcohol or what?. Lancet.

[B40] Benosis A (2004). Social, legal and forensic aspects of alcohol intoxication and mortality.

[B41] McKee M, Sanderson C, Chenet L, Vassin S, Shkolnikov V (1998). Seasonal variation in mortality in Moscow. J Public Health Med.

